# *Chrebp* Deletion and Mild Protein Restriction Additively Decrease Muscle and Bone Mass and Function

**DOI:** 10.3390/nu17030488

**Published:** 2025-01-29

**Authors:** Kanako Deguchi, Chihiro Ushiroda, Shihomi Hidaka, Hiromi Tsuchida, Risako Yamamoto-Wada, Yusuke Seino, Atsushi Suzuki, Daisuke Yabe, Katsumi Iizuka

**Affiliations:** 1 Department of Clinical Nutrition, Fujita Health University, Toyoake 470-1192, Japan; kanasakuran@gmail.com (K.D.); chihiro.ushiroda@fujita-hu.ac.jp (C.U.); risako.wada@fujita-hu.ac.jp (R.Y.-W.); 2 Department of Endocrinology, Diabetes and Metabolism, Fujita Health University, Toyoake 470-1192, Japan; sakai220@fujita-hu.ac.jp (S.H.); seinoy@fujita-hu.ac.jp (Y.S.); aslapin@fujita-hu.ac.jp (A.S.); 3 Department of Diabetes, Endocrinology and Metabolism, Graduate School of Medicine, Gifu University, Gifu 501-1194, Japan; htsuchid@gifu-u.ac.jp (H.T.); ydaisuke@kuhp.kyoto-u.ac.jp (D.Y.); 4 Departments of Diabetes, Endocrinology and Nutrition, Graduate School of Medicine, Kyoto University, Kyoto 606-8507, Japan; 5 Food and Nutrition Service Department, Fujita Health University Hospital, Toyoake 470-1192, Japan

**Keywords:** carbohydrate binding protein, protein, carbohydrate, fat, muscle mass, bone mineral density

## Abstract

**Background/Objectives**: Carbohydrate and protein restriction are associated with sarcopenia and osteopenia, but the underlying mechanisms remain unclear. We aimed to determine whether mild protein restriction affects muscle and bone function in wild-type (WT) and homozygous carbohydrate response element binding protein (*Chrebp*) knockout (KO) mice. **Methods**: Eighteen-week-old male wild-type and homozygous carbohydrate response element binding protein (*Chrebp*) knockout (KO) mice were fed a control diet (20% protein) or a low-protein diet (15% protein) for 12 weeks. We estimated the muscle weight and limb grip strength as well as the bone mineral density, bone structure, and bone morphometry. **Results**: *Chrebp* deletion and a low-protein diet additively decreased body weight (WT control–KO low-protein: mean difference with 95% CI, 8.7 [6.3, 11.0], *p* < 0.0001) and epidydimal fat weight (1.0 [0.7, 1.2], *p* < 0.0001). *Chrebp* deletion and a low-protein diet additively decreased tibialis anterior muscle weight (0.03 [0.01, 0.05], *p* = 0.002) and limb grip strength (63.9 [37.4, 90.5], *p* < 0.0001) due to a decrease in insulin/insulin-like growth factor 1 mRNA and an increase in myostatin mRNA. In contrast, *Chrebp* deletion increased bone mineral density (BMD) (WT control–KO control: –6.1 [–1.0, –2.3], *p* = 0.0009), stiffness (–21.4 [–38.8, –4.1], *p* = 0.011), cancellous bone BV/TV (–6.517 [–10.99, –2.040], *p* = 0.003), and the number of trabeculae (–1.1 [–1.8, –0.5], *p* = 0.0008). However, in KO mice, protein restriction additively decreased BMD (KO control–KO low-protein: 8.1 [4.3, 11.9], *p* < 0.0001), bone stiffness (38.0 [21.3, 54.7], *p* < 0.0001), cancellous bone BV/TV (7.7 [3.3, 12.2], *p* = 0.006), and the number of trabeculae (1.2 [0.6, 1.9], *p* = 0.0004). The effects of mild protein restriction on bone formation parameters (osteoid volume (WT control–WT low-protein: –1.7 [–2.7, –0.7], *p* = 0.001) and the osteoid surface (–11.2 [–20.8, –1.5], *p* = 0.02) were observed only in wild-type (WT) mice. The levels of bone resorption markers, such as the number of osteoclasts on the surface, the number of osteoclasts, and surface erosion, did not differ between the groups. **Conclusions**: Both *Chrebp* deletion and protein restriction led to a decrease in muscle and bone function; therefore, an adequate intake of carbohydrates and proteins is important for maintaining muscle and bone mass and function. Further studies will be needed to elucidate the mechanisms by which ChREBP deletion and a low-protein diet cause osteosarcopenia.

## 1. Introduction

A diversity of food intake is important for maintaining a healthy lifestyle [1]. In particular, the balance of protein, fat, and carbohydrate intake is important [2,3]. In humans, a low protein intake increases the risk of sarcopenia and immune system deficiency, thereby increasing the risk of pneumonia and infection [4,5,6]. An excessive loss of muscle mass is associated with a poor prognosis in several diseases, and significant muscle weakness impairs quality of life [7,8]. Exceptions include diabetic nephropathy and chronic kidney disease, for which a low-protein diet is recommended for management [9,10]. However, in elderly individuals, there are concerns about increasing the risk of sarcopenia when a low-protein diet is recommended, and the recommendation of protein restriction depends on the patient’s condition [11,12,13]. Moreover, several recent epidemiological studies have revealed reduced bone density and increased rates of bone loss in individuals habitually consuming low-protein diets [14,15,16]. Thus, a low-protein diet is associated with an increased risk of muscle atrophy and osteoporosis.

The relationships between low carbohydrate intake and muscle or bone function have been previously reported. With respect to the relationship between carbohydrate intake and muscle function, carbohydrates play a protein-sparing role in the prevention of muscle atrophy [17]. While extreme carbohydrate restriction (e.g., ketogenic diets) may affect bone density, a moderate low-carbohydrate diet may have less of an effect [18]. Therefore, a carbohydrate-restricted diet may negatively affect muscle and bone function.

As a glucose-sensing factor at the transcriptional level, carbohydrate response element binding protein (ChREBP) is known to be a glucose-activated transcription factor [18,19,20]. ChREBP and Max-Ile protein X (MLX) form a heterodimer, and the ChREBP/MLX complex binds to two E boxes in the target gene promoters [19]. The target genes that are positively regulated mainly by ChREBP activation are involved in lipogenesis, gluconeogenesis, glycolysis, the pentose cycle, etc. [19,20,21]. As ChREBP regulates lipogenic gene expression, ChREBP is expressed in the liver, intestines, adipose tissue, pancreatic beta cells, adrenal glands, and macrophages [22,23,24,25,26,27,28,29]. ChREBP is also expressed in muscle [30,31], but the role of ChREBP remains unclear. Moreover, whether ChREBP is expressed in bone remains unclear.

ChREBP is involved in carbohydrate and lipid metabolism but not directly in protein metabolism. However, a combination of protein deficiencies is expected to have a major impact on the organs that metabolize a large amount of energy intracellularly, such as muscle. Furthermore, the skeletal mass index is positively associated with bone mineral density [32]. As weight loss and muscle weakness are expected with *Chrebp* deletion and a low-protein diet, these changes may affect bone density and bone structure.

In this study, we aimed to clarify the role of carbohydrate and protein intake in the prevention of malnutrition-associated muscle atrophy and osteopenia. Using low-protein diet-fed and homozygous *Chrebp* knockout (KO) mice, we tested the effects of protein restriction and *Chrebp* deletion on both muscle and bone mass and function. The use of KO mice is a key feature of this study, as it enables an evaluation of the roles of carbohydrate and protein signaling separately. Compared with a low-protein diet, *Chrebp* deletion decreased muscle weight and limb grip, but *Chrebp* deletion and a low-protein diet additively decreased muscle mass and function. In contrast, *Chrebp* deletion and a low-protein diet had positive and negative effects on bone mineral density and bone stiffness, respectively, but the negative effects of a low-protein diet on bone mineral density and bone stiffness were greater than the positive effects of *Chrebp* deletion. The findings of this study are important for understanding the important role of protein, fat, and carbohydrate balance in maintaining bone and muscle function.

## 2. Materials and Methods

### 2.1. Materials

Lab assay ALP (JAN4548995103048) was purchased from FUJIFILM Wako Pure Chemical Corporation (Osaka, Japan). A mouse/rat insulin kit (catalog no. M1108) was purchased from the Morinaga Institute of Biological Science (Yokohama, Japan). The metalloassay calcium assay LS kit was purchased from Metallogenics Co., Ltd. (Chiba, Japan). The TRACP & ALP Assay Kit was purchased from Takarabio (MK301, Kusatsu, Japan). The PiBlue™ Phosphate Assay Kit was purchased from Bioassay Systems (POPB-500, Hayward, CA, USA).

### 2.2. Animals

The animal experiments were carried out in accordance with the National Institutes of Health Guide for the Care and Use of Laboratory Animals (NIH Publications No. 8023, revised 1978). All animal care was approved by the Fujita Health University Animal Care and Use Committee (APU22030 (2022/1/5), APU22030-MD1 (2022/5/26), and APU22030-MD2 (2023/6/19)), Toyoake, Japan. The mice were housed at 23 °C on a 12-h/12-h light/dark cycle. The mice had free access to water and were fed an autoclaved CE-2 diet (CLEA Japan, Tokyo, Japan). Wild-type (WT) C57BL/6J mice were obtained from SLC (Shizuoka, Japan). Homozygous global *Chrebp*^−/−^ (knockout (KO)) mice were backcrossed for at least 10 generations onto the C57BL/6J background as we previously described [21,22,24]. WT and KO male mice were housed separately, with a total of three mice per cage. The mice had free access to water and were fed an autoclaved CE-2 diet (CLEA Japan, Tokyo, Japan). From 18 to 30 weeks of age, the male mice were fed a control diet (D11112201, Research Diets Inc., Lane New Brunswick, NJ, USA) or a low-protein diet (D22081702, Research Diets Inc.). The percentages of nutrients in the control diets and low-protein diets were as follows: control diet (3.79 kcal/g), 20% protein, 65% carbohydrate, and 15% fat; and low-protein diet (4.06 kcal/g), 15% protein, 70% carbohydrate, and 15% fat. The low-protein diet lacks vitamin D supplementation.

### 2.3. Experiments

**Figure 1:**
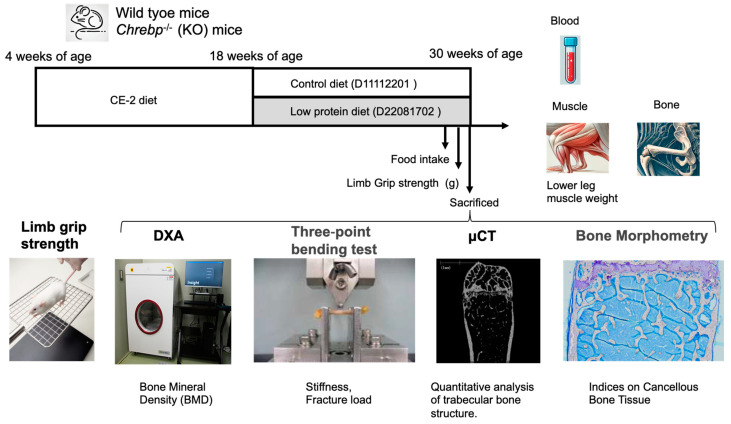
Outline of the experimental design.

At 18 weeks of age, WT and KO mice were fed the control diet or the low-protein diet for 12 weeks (*n* = 15~16 per group). These mice were divided into four groups (control diet-fed WT mice, control diet-fed KO mice, low-protein diet-fed WT mice, and low-protein diet-fed KO mice). After 12 weeks of feeding, blood samples were collected, and the mice were then euthanized by cervical dislocation, and tissue samples were collected (Figure 1). To determine the apparent effect of a low-protein diet on BMD, we considered that the period of the experiment required at least 12 weeks. The tissue samples were snap-frozen in liquid nitrogen and stored at −80 °C until analysis.

### 2.4. Blood Glucose, Insulin, Calcium, Inorganic Phosphate, TRACP, and Alkaline Phosphatase Concentrations Were Measured

For the measurement of the glucose and insulin concentrations, blood samples were obtained from the tail vein of each mouse. The plasma glucose concentrations were measured with a Freestyle Precision Neo kit (Abbott, Chicago, IL, USA). The plasma insulin concentration was measured with a mouse/rat insulin kit (Morinaga Institute of Biological Science, Yokohama, Japan). Amino acid analysis of the blood was performed via the liquid chromatography/mass spectrometry method by SRL CO (Hachiouji, Tokyo, Japan). Alkaline phosphatase activity was measured via a Lab ALP assay (FUJIFILM Wako Pure Chemical Corporation, Osaka, Japan). Tartrate-resistant acid phosphatase (TRACP) activity was measured with a TRACP and ALP Assay Kit (Takarabio, Kusatsu, Japan). The plasma calcium and inorganic phosphate (Pi) concentrations were measured via a metalloassay calcium assay LS kit (Metallogenics Co., Ltd., Chiba, Japan) and a PiBlue™ Phosphate Assay Kit (Bioassay systems, Hayward, CA, USA), respectively.

The WT and KO mice were started on a control (D11112201) or low-protein diet (D22081702) at 18 weeks of age; they were fed a control or low-protein diet from 18 weeks of age and maintained for 12 weeks, after which their organs were harvested and used for experiments. The isolated femurs were used for bone density and bone strength tests, bone structure analysis, and bone morphometry.

### 2.5. Muscle Weight Measurement and Limb Grip Analysis

Muscle weights (tibialis anterior muscle, gastrocnemius muscle, soleus muscle, and extensor digitorum longus muscle) were measured according to previous methods [33].

Limb grip strength tests were performed with a digital force meter (GPM-100B, MELQUEST) [34]. Each mouse was held in the grip, the tail was pulled backward by the hand, and the maximum grip force applied until the grip was released was measured. Each mouse gripped the wire mesh with all four limbs (Figure 1). Grip strength is presented as the average of five measurements per WT or KO mouse at 30 weeks.

### 2.6. Bone Mineral Density Measurement

Bone mineral density measurements were performed at Kureha Special Laboratory (Tokyo, Japan). The bone mineral density was measured with an iNSiGHT noninvasive dual-energy X-ray absorptiometry (DXA) system (OsteoSys Inc., Seoul, Republic of Korea) (Figure 1).

### 2.7. Three-Point Bending Tests

Three-point bending tests are performed on mouse femurs to assess bone strength (the maximum load at which the bone breaks) and flexibility (stiffness) (Figure 1). This test is suitable for long bones, ensuring that the response is predominantly flexural. Three-point bending tests were performed according to previous reports at the Kureha Special Laboratory (Tokyo, Japan) [34]. Femurs were isolated from females at 30 weeks of age, wrapped with Kimwipes dipped in saline, and stored in a –60 °C freezer. After the femurs were thawed, an MZ-500D electromechanical testing machine (Maruto Testing Machine Co., Tokyo, Japan) was used to apply a load vertically to the midshaft with a constant displacement rate of 2 mm/min until fracture was reached. A support span of 6 mm was used.

### 2.8. Microcomputed Tomography Analysis

Quantitative microcomputed tomography (µCT) analysis was performed via a µCT system (Scan Xmate-L090H, Comscantechno Co. Ltd., Yokohama, Japan) at the Kureha Special Laboratory (Tokyo, Japan) (Figure 1) [35]. Data from the scanned slices were used for 3-dimensional analysis to calculate the morphometric parameters of the femurs. Femoral trabecular bone parameters (total bone volume/tissue volume (BV/TV) (%), cancellous bone BV/TV (%), and cortical thickness (μm)) were calculated for the distal femoral metaphysis at 30 weeks of age.

### 2.9. Bone Histomorphometric Analysis

Bone histomorphometric analysis was performed at the Kureha Special Laboratory (Tokyo, Japan) (Figure 1). The femurs of WT and KO mice were harvested and fixed in 70% ethanol for three days. Fixed bones were dehydrated with graded ethanol and infiltrated and embedded in a mixture of methyl methacrylate and 2-hydroxyethyl methacrylate (both from Fujifilm Wako Pure Chemical, Osaka, Japan). Undecalcified 3-μm-thick sections were obtained with a microtome (Leica RM 2255, Leica Biosystems, Nussloch, Germany, GmbH) [35]. Consecutive sections were stained with 0.05% toluidine blue (pH 7.0) for the visualization of osteoid, osteoblasts, and osteoclasts. Bone histomorphometric analysis of the femurs was performed under 200× magnification within a 0.75 mm high × 0.7 mm wide region located 300 μm from the growth plate with Bone Histometry RT camera analysis software (version: Bone60 v1.26b, System-Supply, Nagano, Japan). Twenty-micron cross sections from the mid-diaphyses of the femurs were used for the bone histomorphometric analysis of cortical bone. The structural, dynamic, and cellular parameters were calculated and expressed according to the standard nomenclature [35]. The following parameters were measured: structural parameters ((BV/TV), trabecular number (Tb.N), and trabecular separation), bone formation parameters (osteoid volume/bone volume (OV/BV), and the osteoid surface/bone surface (OS/BS), osteoblast surface/bone surface (Ob.S/BS), and osteoid thickness (O.Th)), and bone resorption markers (osteoclast surface/bone surface (Oc.S/BS), osteoclast number/bone surface (N.Oc/BS), and eroded surface/bone surface (ES/BS)).

### 2.10. RNA Isolation and Quantitative Reverse-Transcription PCR

The mRNA isolation, cDNA synthesis, and quantitative reverse-transcription PCR (qRT–PCR) analysis were performed as previously described [23]. Briefly, RNA was extracted from 100 mg of frozen liver, muscle, or proximal femur pooled from three mice per sample with an RNeasy Plus Universal Kit (Qiagen, Hilden, Germany). Femur and muscle were milled in a mortar under liquid nitrogen before RNA extraction. cDNA was synthesized with a high-capacity cDNA Reverse Transcription Kit (Thermo Fisher Scientific, Waltham, MA, USA). Gene expression levels were evaluated in three liver, muscle, and bone tissue samples. The relative quantification of the mRNA levels was performed with reference to a standard curve. RNA polymerase 2 (*Pol2*) was used as the reference gene. Real-time PCR was performed with an ABI PRISM9700 (Thermo Fisher Scientific, Waltham, MA, USA). The primers used for RT–PCR are listed in Table 1.

### 2.11. Statistical Analysis

The values are expressed as the means ± standard deviations (SDs). The exclusion of individual data points was determined via an outlier calculator included in the Prism 10 software package (GraphPad Software Inc., San Diego, CA, USA) and excluded from the analyses. Samples with obviously abnormal laboratory values (below the sensitivity or above the limit of measurement) and samples with severe hemolysis were excluded from the examination. Mice with severe skin alopecia, emaciated mice, and mice with masses or other abnormalities were also excluded from the experimental group. Statistical analysis was performed via one-way ANOVA followed by Tukey post hoc tests. A *p* value < 0.05 was considered statistically significant. Sample sizes were based on previous experimental experience in our research group and on previous studies in the literature.

## 3. Results

### 3.1. Chrebp Is Expressed in Skeletal Muscle and Bone

ChREBP is known to be expressed in lipogenic tissues such as liver, intestine, and adipose tissues. Given that sarcopenia and osteoporosis are representative phenomena in muscle and bone caused by malnutrition, we first determined *Chrebp* mRNA levels in skeletal muscle and bone tissues.

**Figure 2:**
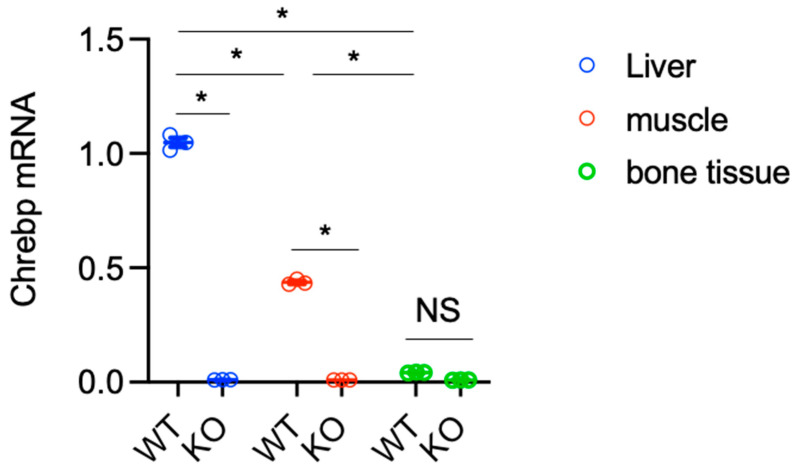
Distribution of carbohydrate response element binding protein (*Chrebp*) mRNAs. * *p* < 0.05. NS: not significant.

The *Chrebp* mRNA levels in the skeletal muscle and bone tissues of WT mice were one-half and one-twenty times greater than those in the liver. In contrast, *Chrebp* mRNA was not detected in the liver, skeletal muscle, or bone tissues of the KO mice. Thus, *Chrebp* mRNA is expressed in the liver (WT: 1.05 ± 0.03; KO: 0.010 ± 0.0003, *p* < 0.001), skeletal muscle (WT: 0.45 ± 0.01; KO: 0.001 ± 0.0001, *p* < 0.001) and, to a lesser extent, bone tissues (WT: 0.041 ± 0.001; KO: 0.009 ± 0.00002, *p* = 0.13) (Figure 2).

The liver, anterior tibialis muscle, and femur heads were crushed in liquid nitrogen, and RNA was subsequently extracted from the liver, muscle, and bone tissues of wild-type (WT) and homozygous carbohydrate response element (*Chrebp)* knockout (KO) mice. The mRNA levels were normalized to those of mouse *Pol2* mRNA. The data are presented as the means ± standard error of the means, *n* = 3. 

### 3.2. Low-Protein Diet-Fed Chrebp KO Mice Presented Lower Body Weights and Muscle Masses

*Chrebp* KO mice are known to have decreased epidydimal fat weight and increased liver weight due to a decrease in de novo lipogenesis and hepatic glycogen accumulation. However, the effects of a low-protein diet on KO mice remain unknown.

**Figure 3:**
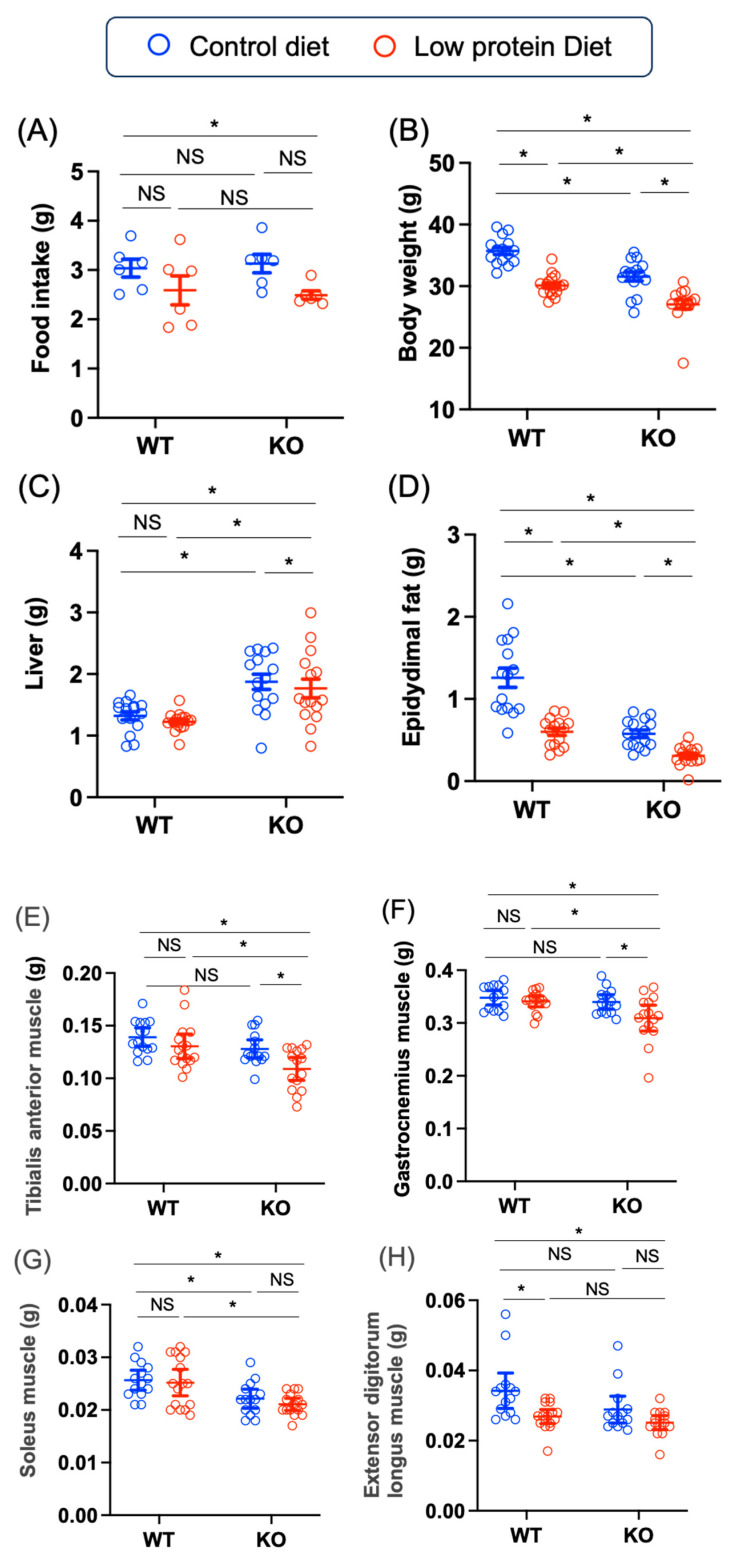

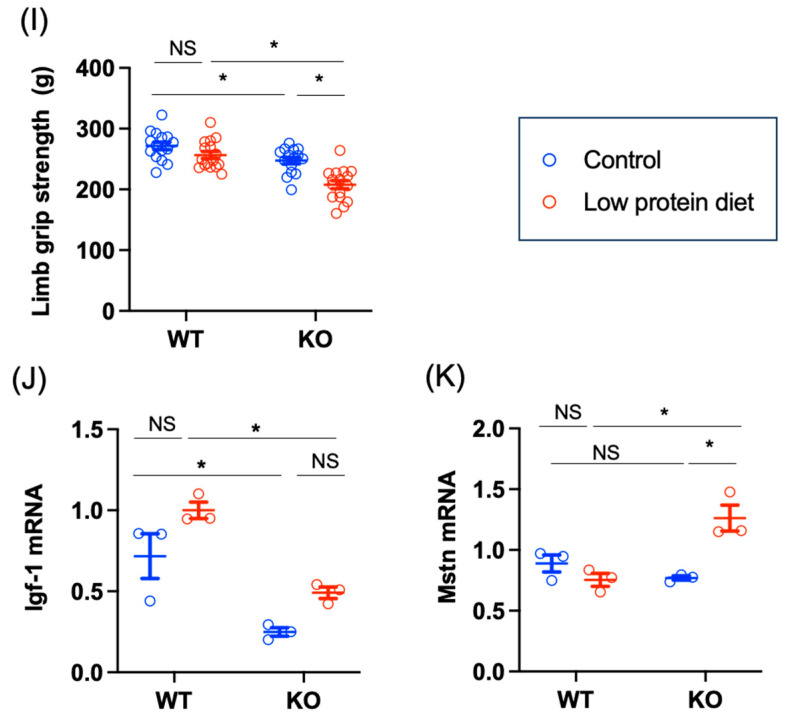
Effects of *Chrebp* deletion and low-protein diet feeding on body weight and tissue weight. (**A**) The amount of food intake (grams per day) was *n* = 6 for each group (control diet: blue; low-protein diet: red). (**B**) total body weight (grams), (**C**) liver weight (grams), (**D**) epididymal fat weight (g), (**E**) tibialis anterior (TA) muscle weight (g), (**F**) gastrocnemius muscle weight (g), (**G**) soleus muscle weight (g), (**H**) extensor digitorum longus muscle weight (g), and (**I**) limb grip strength (g), *n* = 14~16 for each group (control diet: blue; low-protein diet: red). After 12 weeks of rearing on a normal or low-protein diet, the tissues were removed and weighed. Muscle isolation was performed according to previous reports [32]. The data are presented as the means ± standard error of the means (SEMs) and were analyzed via one-way analysis of variance (ANOVA) with post hoc Tukey’s test for each group (control diet: blue; low-protein diet: red). * *n* = 14~16, *p* < 0.05. (**J**) muscle insulin-like growth factor (*Igf-1*) mRNA, (**K**) muscle myostatin (*Mstn*) mRNA. The anterior tibialis muscles were crushed under liquid nitrogen. mRNA was extracted from the muscle, and cDNA was synthesized as described in the Materials and Methods. *Igf-1* and *Mstn* mRNA levels were measured via real-time PCR methods. The data are presented as the means ± standard error of the means ± SEMs and were analyzed via one-way ANOVA with post hoc Tukey’s test for each group (control diet: blue; low-protein diet: red). * *n* = 3, *p* < 0.05. NS; not significant.

In this study, the energy levels in the low-protein diets were relatively similar (control diet 3.8 kcal/g and low-protein diet 4.1 kcal/g), whereas the protein contents were much lower (control 20% and low-protein diet 15%). We first tested whether a low-protein diet influenced body size in WT and KO mice. In the control diet-fed groups, food intake was similar between the WT and KO mice. Low-protein diets were also used. The food intake for the WT and KO mice was similar, although that of the low-protein diet-fed mice tended to decrease (Figure 3A).

In terms of body weight, low-protein diet feeding led to a lower body weight in both WT and KO mice than did control diet feeding, and the body weights of the KO mice fed the low-protein diet were the lowest among the control and low-protein diet-fed WT and KO mice (WT control–KO low-protein: mean difference [95% CI], 8.65 [6.3, 11.0], *p* < 0.0001) (Figure 3B). In terms of liver weight, the liver weights of the KO mice fed the control diet were greater than those of the WT mice fed the control diet (WT control–KO control: –0.5 [–0.9, –0.2], *p* = 0.002), which is consistent with the findings of our previous paper. In addition, the liver weights of the KO mice fed the low-protein diet were greater than those of the WT mice fed the low-protein diet (WT low-protein–KO low-protein:–0.5 [–0.9, –0.2], *p* = 0.003); however, the liver weights of the KO mice fed the low-protein diet were lower than those of the KO mice fed the control diet (*p* = 0.88) (Figure 3C). With respect to epidydimal fat weights, both *Chrebp* deletion and low-protein diet feeding led to a decrease in epidydimal fat weights, and the epidydimal fat weights of the KO mice fed the low-protein diet were the lowest (WT control–KO low-protein: 1.0 [0.7, 1.2], *p* < 0.0001) (Figure 3D). Thus, the KO mice presented with decreased adiposity and increased liver weight, and a low-protein diet promoted decreased body weight and adiposity.

With respect to muscle mass, low-protein diet feeding did not lead to a decrease in tibialis anterior (TA) muscle weight in WT mice (WT control–WT low-protein: 0.08 [–0.01, 0.03], *p* = 0.68); however, the TA weight in KO mice fed the low-protein diet was lower than that in KO mice fed the control diet (KO control–KO low-protein: 0.02 [0.0009, 0.03], *p* = 0.04) (Figure 3E). Moreover, the TA, gastrocnemius, soleus, and extensor digitorum longus muscle weights of the KO mice fed the low-protein diet were significantly lower than those of the WT mice fed the control diet (WT control–KO low-protein: TA 0.03 [0.01, 0.05], *p* = 0.0009; gastrocnemius 0.04 [0.01, 0.07], *p* = 0.005; soleus muscle 0.005 [0.001, 0.008], *p* = 0.004; extensor digitorum longus muscle 0.009 [0.003, 0.01], *p* = 0.0008) (Figure 3E–H). Consistent with these findings, the limb grip strength of the KO mice fed the low-protein diet was lower than that of the KO mice fed the control diet (KO control–KO low- protein: 39.7 [22.5, 56.9], *p* < 0.0001) and the WT mice fed the low-protein diet (WT low- protein diet–KO low-protein diet: 48.7 [28.3, 69.2], *p* < 0.0001) (Figure 3F). In terms of muscle-related mRNAs, insulin/insulin-like growth factor 1 (*Igf-1*) mRNA levels were lower in KO mice than in WT mice, independent of diet (WT control–KO control: 0.5 [0.09, 0.8], *p* = 0.02; WT low-protein–KO low-protein: 0.5 [0.1, 0.9], *p* = 0.0095) (Figure 3G). In contrast, the myostatin (*Mstn*) mRNA level in the KO mice fed the low-protein diet was greater than that in the KO mice fed the control diet (KO control–KO low-protein: –0.5 [–0.8, –0.2], *p* = 0.005) and WT mice fed the low-protein diet (WT low-protein–KO low-protein: –0.5 [–0.8, –0.2], *p* = 0.004) (Figure 3H). Thus, *Chrebp* deletion and a low-protein diet additively decreased muscle weight and limb grip strength.

### 3.3. Chrebp Deletion and Mild Protein Restriction Increased Plasma Alanine and Glutamine Levels

**Figure 4:**
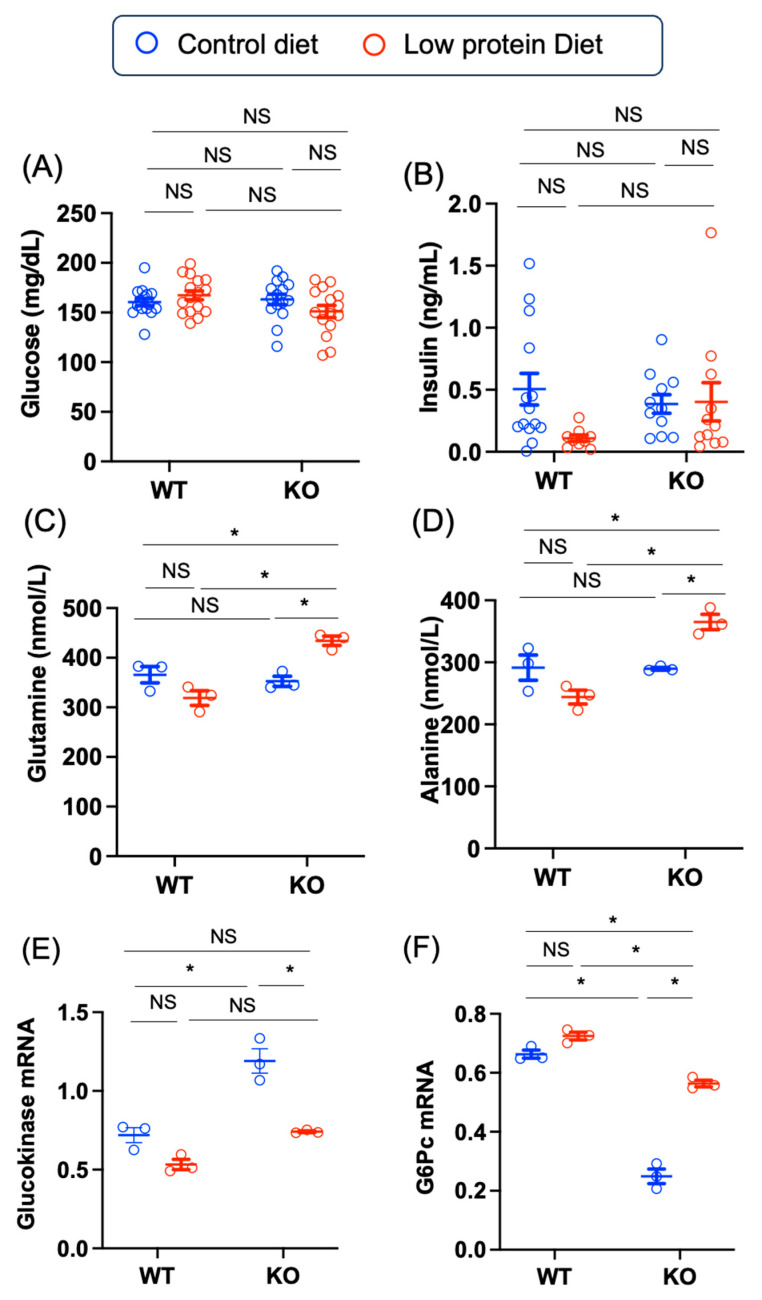
Effects of Chrebp deletion and low-protein diet feeding on glucose, insulin, and amino acid levels. (**A**) plasma glucose (mg/dL). The data are presented as the means ± the means ± standard error of the means (SEMs) and were analyzed via one-way analysis of variance (ANOVA) with post hoc Tukey’s test for each group. *n = 15~16, *p* < 0.05. (**B**) insulin (ng/mL) levels. The data are presented as the means ± SEMs and were analyzed via one-way analysis of variance (ANOVA) with post hoc Tukey’s test for each group (control diet: blue; low-protein diet: red). * *n* = 9~14, *p* < 0.05. (**C**) plasma glutamine (nmol/L) and (**D**) plasma alanine (nmol/L) levels. The data are presented as the means ± SEMs and were analyzed via one-way ANOVA with post hoc Tukey’s test for each group (control diet: blue; low-protein diet: red). * *n* = 3, *p* < 0.05. (**E**) liver glucokinase (*Gck*) and (**F**) liver glucose-6-phosphatase catalytic subunit (*G6pc*) mRNA levels. mRNA was extracted from muscle, and cDNA was synthesized as described in the Materials and Methods. Liver *Gck* and *G6pc* mRNA levels were measured via real-time PCR methods. The data are presented as the means ± SEMs and were analyzed via one-way ANOVA with post hoc Tukey’s test for each group (control diet: blue; low-protein diet: red). * *n* = 3, *p* < 0.05. NS: not significant.

The reduced muscle strength and muscle weight in the KO mice fed a low-protein diet suggested that muscle breakdown and the release of amino acids may have occurred in these mice. Therefore, we measured several amino acids along with blood glucose and insulin in WT and KO mice. The plasma glucose, glucose, and insulin levels were similar among the four groups (control diet-fed WT mice, control diet-fed KO mice, low-protein diet-fed WT mice, and low-protein diet-fed KO mice) (Figure 4A,B). However, the plasma glutamine and alanine levels derived from muscle were highest in the KO mice fed the low-protein diet (glutamine: –68.3 [–127.3, –9.4], *p* = 0.02; alanine: –73.7 [–133.6, –13.8], *p* = 0.02) (Figure 4C,D). Consistent with these findings, hepatic glucokinase (*Gck*) mRNA levels in the KO mice were suppressed by low-protein diet feeding (KO control—KO low-protein: 0.4 [0.2, 0.7], *p* = 0.0008) (Figure 4E). Consistent with the fact that *G6pc*, a gluconeogenic gene, is positively regulated by Chrebp, glucose-6-phosphatase catalytic subunit (*G6pc*) mRNA levels were lower in the KO mice than in the WT mice (WT control–KO control: 0.4 [0.3, 0.5], *p* < 0.0001) (Figure 4F). However, a low-protein diet led to an increase in hepatic *G6pc* mRNA levels in the KO mice via the maintenance of plasma glucose levels (KO control—KO low-protein; –0.3 [–0.4, –0.2], *p* < 0.0001). Thus, low-protein diet-fed KOs presented with increased hepatic gluconeogenesis due to the increased supply of amino acids (alanine and glutamine) from muscle.

### 3.4. Chrebp Deletion and a Low-Protein Diet Positively and Negatively Affect BMD and Stiffness, Respectively

Bone mineral density is also known to decrease in malnutrition. We next evaluated bone mineral density and strength as well as calcium and phosphate levels.

**Figure 5:**
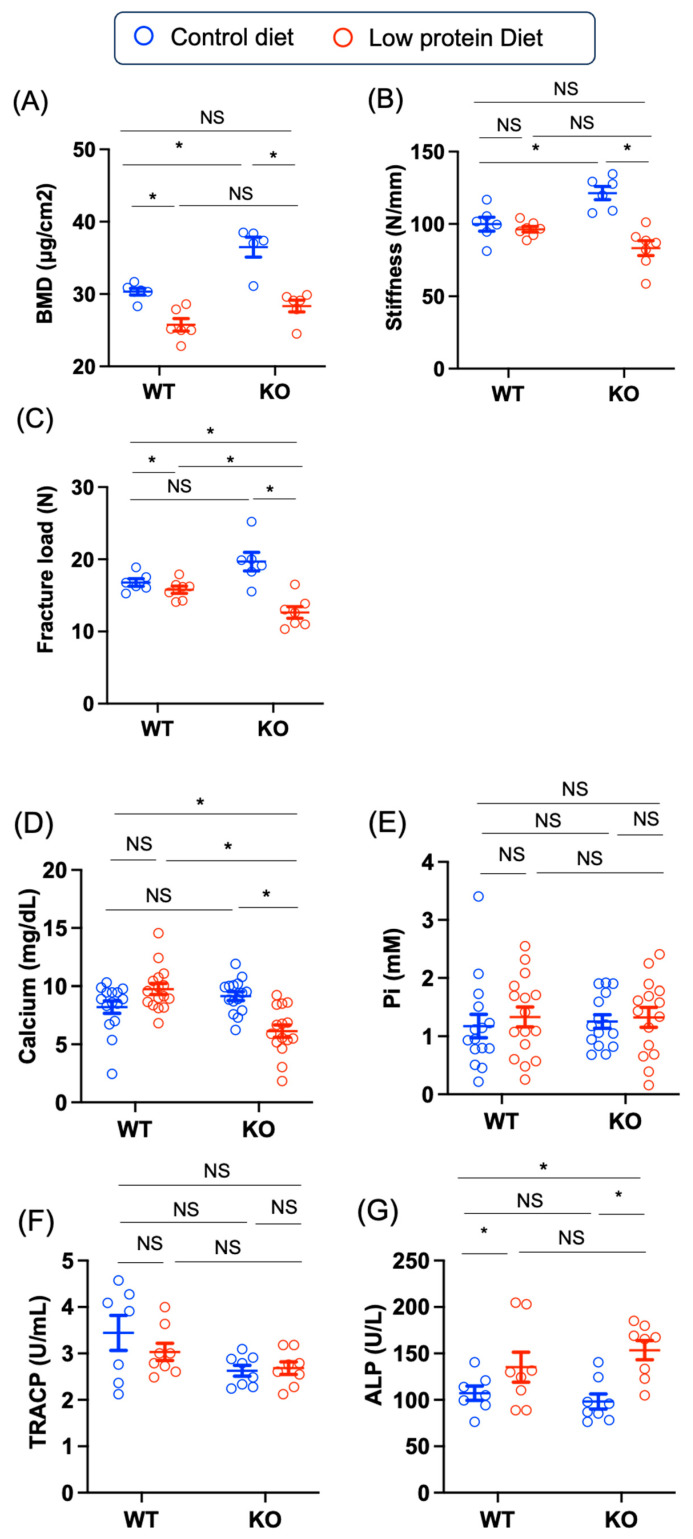
Effects of Chrebp deletion and low-protein diet feeding on BMD and stiffness. (**A**) bone mineral density. The bone mineral density of the femur was measured via noninvasive dual-energy X-ray absorptiometry (DXA). The data are presented as the means ± standard error of the means (SEMs) and were analyzed via one-way analysis of variance (ANOVA) with post hoc Tukey’s test for each group (control diet: blue; low-protein diet: red). * *n* = 6, *p* < 0.05. (**B**) stiffness (N/cm^2^) and (**C**) fracture load (N). The stiffness and fracture load of the femur were measured via three-point bending tests. The data are presented as the means ± SEMs and were analyzed via one-way ANOVA with post hoc Tukey’s test for each group. *n = 6, *p* < 0.05. (**D**) plasma calcium levels and (**E**) plasma phosphate levels. The data are presented as the means ± SEMs and were analyzed via one-way ANOVA with post hoc Tukey’s test for each group. * *n* = 15~16, *p* < 0.05. (**F**) plasma TRACP levels (U/mL) and (**G**) plasma alkaline phosphatase (ALP) levels. The data are presented as the means ± SEMs and were analyzed via one-way ANOVA with post hoc Tukey’s test for each group (control diet: blue; low-protein diet: red). * *n* = 7~8, *p* < 0.05. NS: not significant.

In terms of bone mineral density, the BMD in the KO group was greater than that in the WT group under control diet conditions (WT control—KO control: –6.1 [–9.8, –2.5], *p* = 0.0008). Low-protein diet feeding led to a decrease in the BMD in both WT and KO mice (WT control—WT low-protein: –6.1 [–9.8, –2.5], *p* = 0.0008; KO control—KO low-protein: 8.1 [4.5, 11. 8], *p* < 0.0001), and the BMDs were similar between the WT and KO mice fed a low-protein diet (WT low-protein—KO low-protein: –2.6 [–6.1, 0.9], *p* = 0.19) (Figure 5A). Next, we performed a three-point bending test. The results regarding dynamic stiffness were almost compatible with those of BMD; the stiffness in the KO mice fed the control diet was greater than that in the WT mice fed the control diet (WT control—KO control: –21.4 [–38.8, –4.1], *p* = 0.011), and that in the KO mice fed the low-protein diet (KO control—KO low-protein: 38.0 [21.3, 54.7], *p* < 0.0001) (Figure 5B). The fracture load in KO mice fed the low-protein diet was lower than that in WT mice fed the low-protein diet (WT low-protein–KO low-protein: 3.1 [0.06, 6.3], *p* = 0.045) and KO mice fed the control diet (KO control–KO low-protein: 7.0 [3.8, 10.3], *p* < 0.0001) (Figure 5C). Thus, *Chrebp* deletion and a low-protein diet have opposite effects on BMD and bone stiffness.

The plasma calcium levels in the KO mice fed the low-protein diet were the lowest (WT control–KO low-protein: 2.1 [0.2, 3.9], *p* = 0.02) (Figure 5D), and the plasma inorganic phosphate levels were similar among the groups (Figure 5E). The plasma TRACP levels were similar (Figure 5F); however, the plasma ALP levels were increased in low-protein diet-fed KO mice compared with their control diet-fed counterparts (KO control–KO low-protein: –55.3 [–98.3, –12.3], *p* = 0.008) (Figure 5G). Thus, a low-protein diet decreases blood calcium levels only in KO mice.

### 3.5. Chrebp Depletion and Low-Protein Diet Feeding Have Opposite Effects on Bone Structure

**Figure 6:**
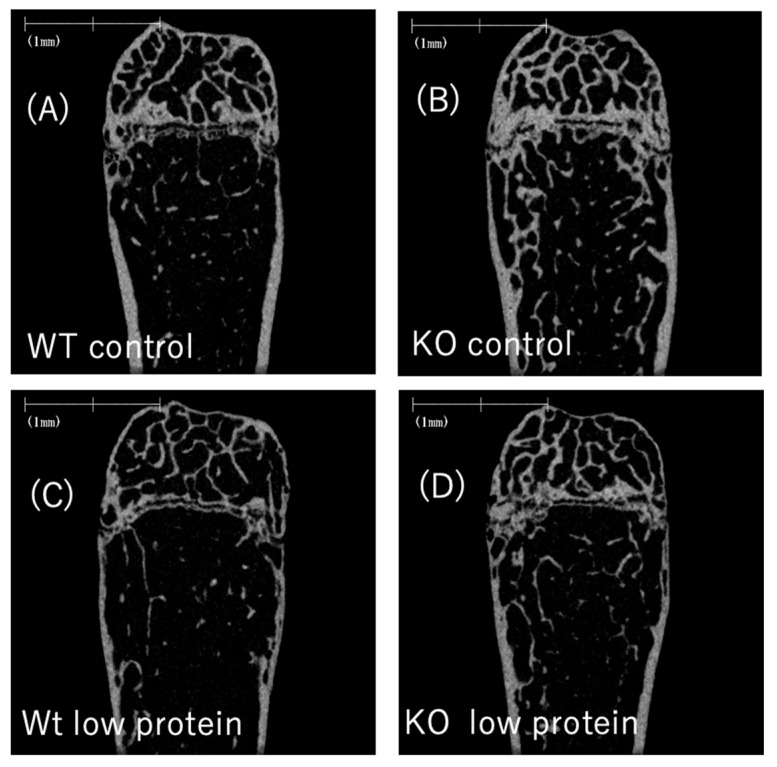

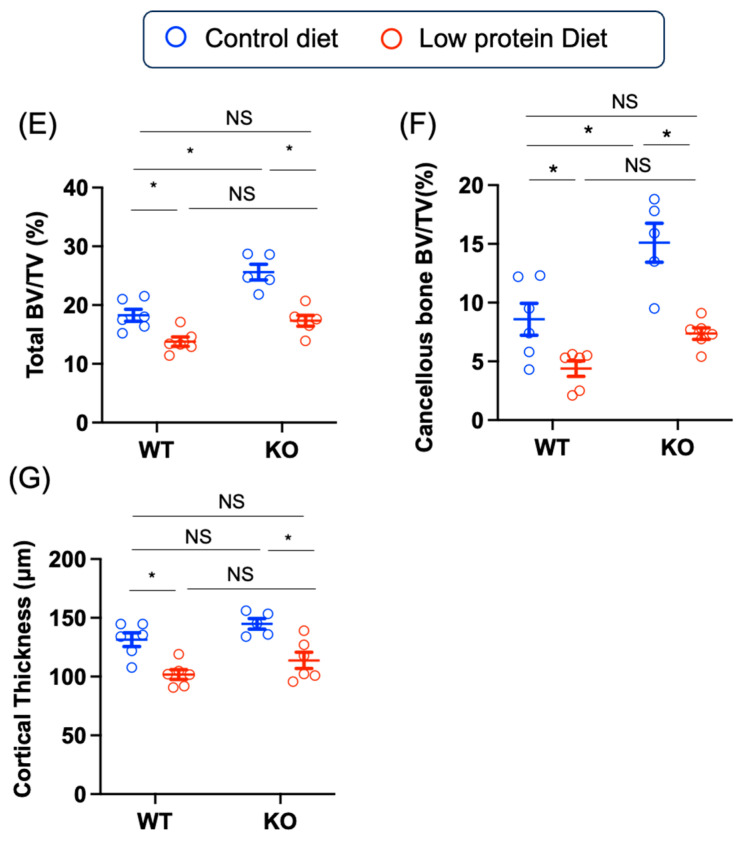
Effects of Chrebp deletion and a low-protein diet on bone structure parameters. Micro-CT images of (**A**) wild-type (WT) control diet-fed, (**B**) knockout (KO) control diet-fed, (**C**) WT low-protein diet-fed, and (**D**) KO low-protein diet-fed mice. (**E**) total bone volume per total volume BV/TV (%), (**F**) cancellous bone BV/TV (%), and (**G**) cortical thickness (μm). BV/TV, cancellous bone BV/TV, and cortical thickness were measured via micro-CT. The data are presented as the means ± standard error (SEMs) and were analyzed via one-way analysis of variance (ANOVA) with post hoc Tukey’s test for each group (control diet: blue; low-protein diet: red). * *n* = 5–6, *p* < 0.05. The data are presented as the means ± SEMs. NS: not significant.

Bone is composed of trabecular bone and cortical bone. Trabecular bone is the inner part of the bone and is similar to a beam (called a bony beam). Cortical bone, on the other hand, is the outer part of the bone and comprises very hard tissue. Trabecular bone has a larger surface area than cortical bone and is more susceptible to changes in bone metabolism because it contains more osteoclasts and osteoblasts, which are responsible for bone metabolism. To determine the effects of *Chrebp* deletion and low-protein diet feeding, we next performed μCT examination and trabecular bone structure analysis (Figure 6A–D). The μCT revealed that *Chrebp* deletion and a low-protein diet led to an increase and a decrease in the bone volume per total tissue volume (BV/TV), respectively, in both WT and KO mice (total cancellous BV/TV), which was consistent with the BMD results (Figure 6E–G). The total and cancellous BV/TV ratios in the KO mice fed the low-protein diet were the same as those in the WT and KO mice fed the low-protein diet (total; cancellous BV/TV). In contrast, the cortical thickness in control diet-fed KO mice was similar to that in control diet-fed WT mice (WT control−KO control: −13.46 [−36.2, 9.3], *p* = 0.37). However, low-protein diet feeding similarly led to a decrease in the cortical thickness in WT and KO mice (WT control−WT low-protein diet: 29.8 [8.0, 51.5], *p* = 0.005; KO control−WT low-protein diet: 31.1 [8.4, 53.9], *p* = 0.006). Thus, the effect of *Chrebp* gene deletion on bone volume was observed only in cancellous bone susceptible to changes in bone metabolism.

**Figure 7:**
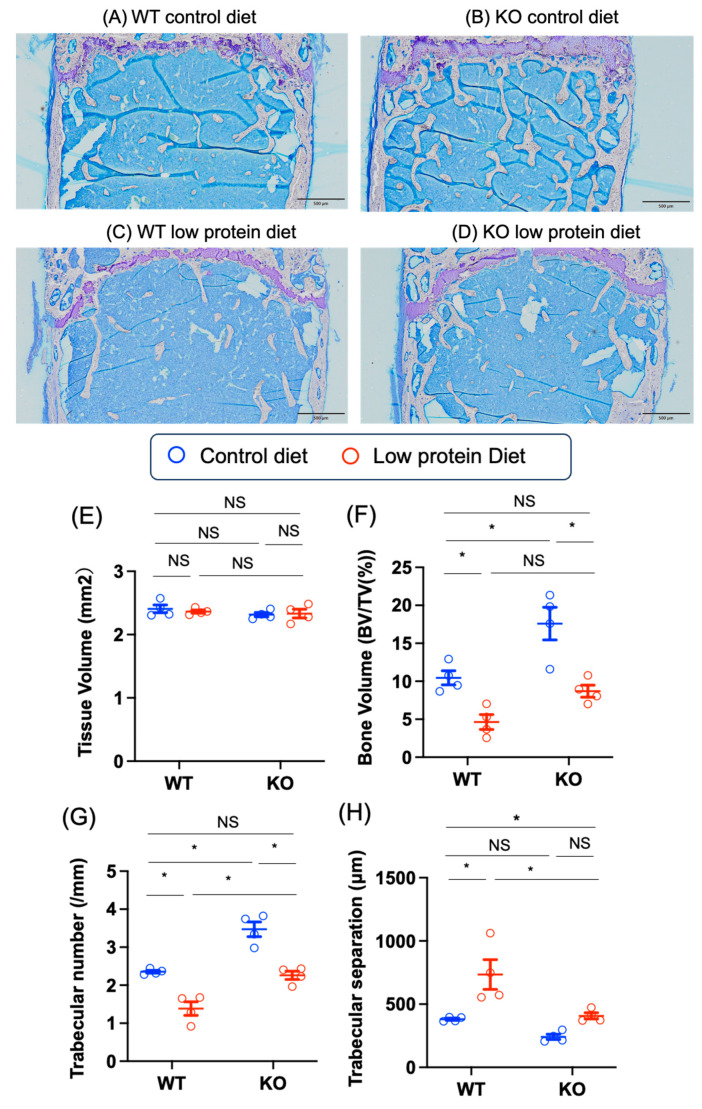

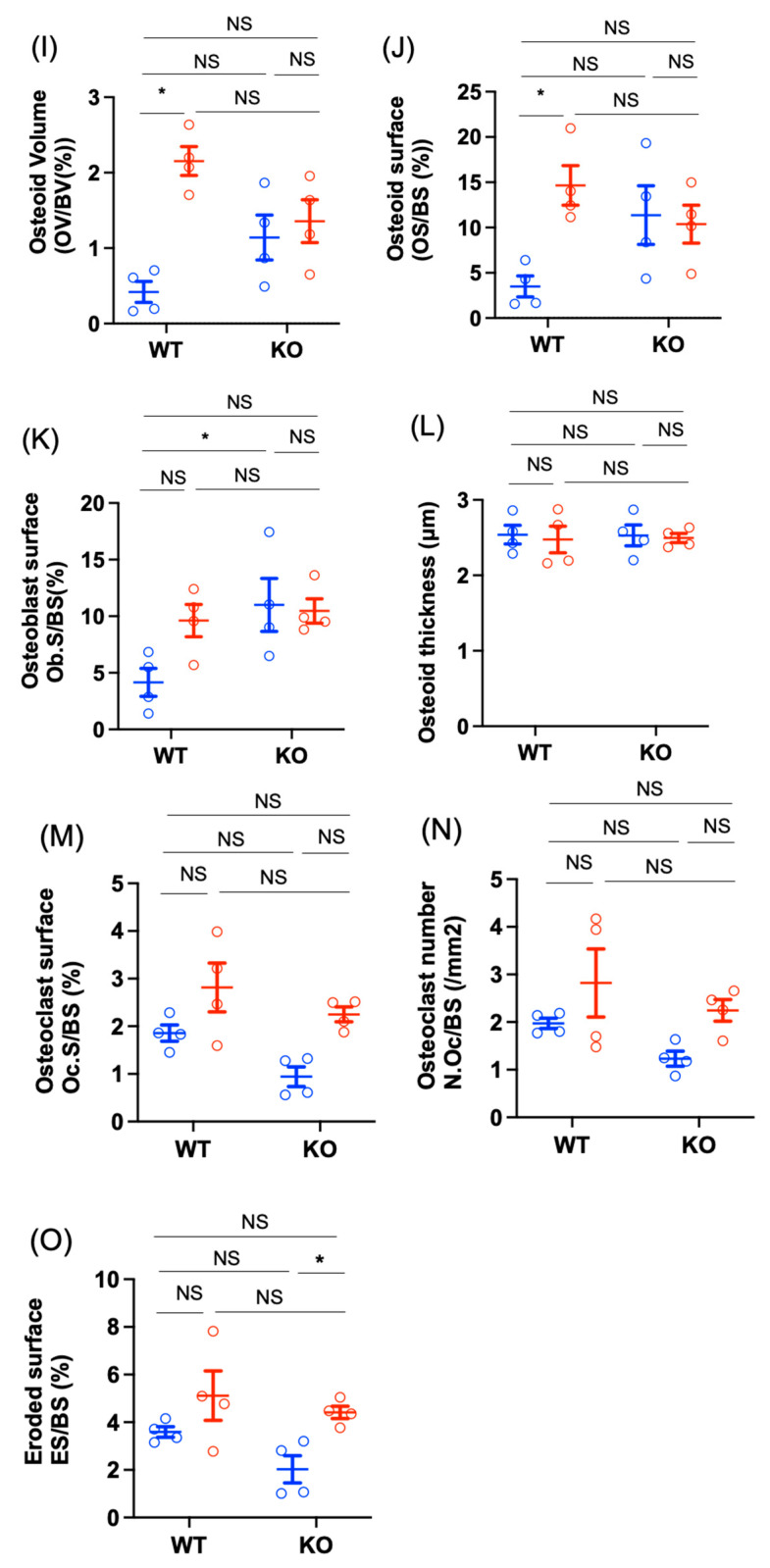
Effects of Chrebp deletion and low-protein diet feeding on bone structure, bone formation, and bone resorption. Representative images (magnification: 40×) of femur epiphyses of (**A**) the wild-type (WT) control diet-fed, (**B**) knockout (KO) control diet-fed, (**C**) WT low-protein diet-fed, and (**D**) KO low-protein diet-fed groups. (**E**) bone volume (BV/TV (%)), (**F**) number of trabeculae (mm), (**G**) trabecular separation (µm), (**H**) osteoid volume (OV/BV) (%), (**I**) osteoid surface (%), (**J**) osteoblast surface (%), (**K**) osteoid thickness (µm), (**L**) osteoclast surface (%), (**M**) osteoclast number (/mm^2^), and (**N**) osteoclast surface, and (**O**) surface erosion EB/BS (%). The structural, dynamic, and cellular parameters were calculated and expressed according to the standard nomenclature [35]. The data are presented as the means ± standard error of the means and were analyzed via one-way analysis of variance (ANOVA) with post hoc Tukey’s test for each group (control diet: blue; low-protein diet: red). * *n* = 4, *p* < 0.05. NS: not significant.

Next, we performed trabecular bone morphometric analysis (Figure 7A–O). In the control diet group, the bone volume (BV/TV (%)) and the number of trabeculae (mm) in the KO group were greater than those in the WT group (BV/TV WT control–KO control: −7.1 [−12.7, −1.6], *p* = 0.011; number of trabeculae WT control–KO control: −1.1 [−1.8, −0.5], *p* = 0.0008). Low-protein diet feeding led to a decrease in the bone volume (WT control—WT low-protein: 5.8 [0.2, 11.4], *p* = 0.04]; KO control—KO low-protein: 9.0 [3.3, 14.5], *p* = 0.002) and the number of trabeculae (WT control—WT low-protein: 1.0 [0.3, 1.6], *p* = 0.003; KO control—KO low-protein: 1.2 [0.6, 1.9], *p* = 0.0004) in both WT and KO mice (Figure 7A–G). Consistent with these findings, the degree of trabecular separation in the KO group was lower than that in the WT group under low-protein diet conditions (WT low-protein—KO low-protein: 326.6 [55.5, 597.7], *p* = 0.02) (Figure 7H).

In terms of bone formation, the osteoid volume and surface area in WT mice fed the low-protein diet were greater than those in WT mice fed the control diet (osteoid volume WT control—WT low-protein: –1.7 [–2.7, –0.7], *p* = 0.001; osteoid surface WT control—WT low-protein: –11.2 [–20.8, –1.5], *p* = 0.02), whereas the osteoid volume and surface area were similar in KO mice fed the control diet and the low-protein diet (osteoid volume KO control—KO low-protein: –0.2 [–1.2, 0.8], *p* = 0.92) (Figure 7I,J). Compared with that in WT mice fed the control diet, the osteoblast surface area in KO mice fed the control diet was greater (WT control—KO control: –6.8 [–13.6, –0.1], *p* = 0.045), but low-protein diet feeding did not affect the osteoblast surface area in KO mice (KO control—KO low-protein: 0.5 [–6.2, 7.2], *p* = 0.99) (Figure 7K). Osteoid thickness was similar among the groups (Figure 7L). In terms of bone absorption, the number of osteoclasts, the number of osteoblasts, and surface erosion were similar among the groups, although the low-protein diet tended to lead to an increase in bone absorption, the number of osteoclasts, the number of osteoblasts, and surface erosion (Figure 7M–O).

**Figure 8:**
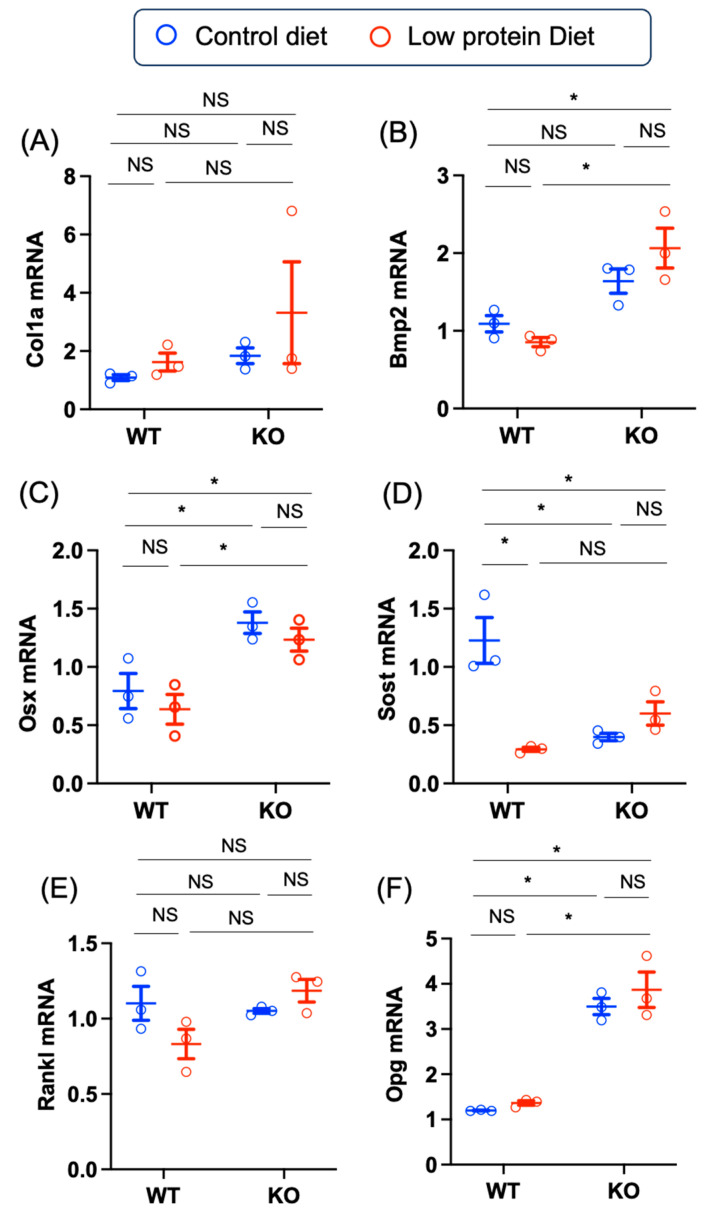
Effects of Chrebp deletion and low-protein diet feeding on bone formation and resorption-related mRNA expression. (**A**) *Col1a* mRNA, (**B**) *Bmp2* mRNA, (**C**) *Osx* mRNA, (**D**) *Sost* mRNA, (**E**) *Rankl* mRNA, (**F**) Opg mRNA. Femoral heads from WT and KO mice fed a normal or low-protein diet for 12 weeks were isolated and ground under liquid nitrogen, RNA was isolated, and cDNA was synthesized. The mRNA levels were normalized to those of mouse *Pol2* mRNA. The data are presented as the means ± standard error of the means and were analyzed via one-way analysis of variance (ANOVA) with post hoc Tukey’s test for each group (control diet: blue; low-protein diet: red). * *n* = 3, *p* < 0.05. NS: not significant.

Finally, we evaluated mRNAs related to bone formation and absorption. The level of *Col1a* mRNA tended to be highest in the low-protein diet-fed KO mice (Figure 8A). As Chrebp deletion tended to increase the expression of bone morphogenetic protein-2 (*Bmp2*) and the osteoblast-specific transcription factor Osterix (*Osx*) mRNAs, the expression of *Bmp2* and *Osx* mRNAs was highest in low-protein diet-fed KO mice (*Bmp2* WT control—KO low-protein: –1.2 [–1.9, −0.5], *p* = 0.003; *Osx* WT control—KO low-protein: –0.6 [–1.0, −0.2], *p* = 0.008) (Figure 8B,C). In contrast, sclerotin (*Sost*) mRNA levels were highest in the control diet-fed WT group (WT control—KO low-protein: 0.6 [0.1, 1.1], *p* = 0.02) (Figure 8D). In terms of bone absorption, receptor activator of nuclear factor kappa-Β ligand (*Rankl*) mRNA levels were similar, but osteoprotegerin (*Opg*) mRNA levels were greater in KO mice fed both the control diet and the low-protein diet (Opg WT control—KO control: –2.3 [–3.3, –1.3], *p* = 0.0003; WT control—KO low-protein:–2.7 [−3.6, −1.7], *p* = 0.0001) (Figure 8E,F). Thus, *Chrebp* deletion tends to promote bone formation rather than bone absorption.

## 4. Discussion

The restriction of carbohydrates as well as protein during starvation is known to lead to skeletal muscle loss, but the mechanism is unknown. To elucidate some of the mechanisms of protein and carbohydrate restriction in muscle and bone, we evaluated whether *Chrebp* deletion and low-protein diet feeding promote malnutrition, especially in muscle and bone tissues. A low-protein diet itself did not significantly affect muscle weight or limb grip strength but did lead to a decrease in bone mineral density in WT mice. In contrast, *Chrebp* deletion itself led to a decrease in muscle limb grip strength and an increase in bone mineral density and stiffness. Interestingly, the combination of a low-protein diet and *Chrebp* deletion led to decreases in muscle weight, limb grip strength, and bone mineral density and stiffness. Thus, the suppression of *Chrebp* has the opposite effect on decreasing muscle strength and increasing bone density. When protein is restricted, both bone density and muscle mass decrease. Nutritional guidance should be provided to ensure that both protein and carbohydrates are not depleted to maintain skeletal muscle and bone mass.

In this study, *Chrebp* mRNA was detected in both muscle and bone tissues. We previously reported that *Chrebp* mRNA is expressed in muscle [21]; however, we first reported that *Chrebp* is also expressed in bone tissues. Attempts to localize ChREBP to bone by immunostaining were unsuccessful, probably due to low expression levels. We could not determine whether *Chrebp* mRNA is expressed in osteoclasts or osteoblasts. Findings from gene expression studies have suggested that *Chrebp* deletion promotes osteogenesis, but findings from other studies have suggested that *Chrebp* is expressed in macrophages [35,36,37,38]. Considering that osteoclasts develop from the monocyte/macrophage lineage, *Chrebp* may be expressed in osteoclasts or bone marrow. Since there are cultured cell lines of osteoblasts and osteoclasts, it is necessary to clarify whether the expression of *Chrebp* in osteoblasts and osteoclasts changes with the differentiation of each cell.

In both the control diet and low-protein diet groups, *Chrebp* deletion led to a decrease in total body weight, epidydimal fat weight, and muscle weight but an increase in liver weight. Increased liver weights and decreased white adipose tissue (WAT) weights are characteristic of KO mice because of increased hepatic glycogen contents and decreased de novo lipogenesis in WAT. The food intake in the low-protein diet tended to be lower than that in the control diet, but the energy per gram in the low-protein diet (3.7 kcal/g) was slightly greater than that in the control diet (4.1 kcal/g). The total energy in the control and low-protein diet groups was similar, and an imbalance in the protein–fat–carbohydrate ratio may have led to decreases in body weight, epidydimal fat weight, and muscle weight.

*Chrebp* deletion led to a decrease in muscle mass and limb grip strength, which was compatible with the changes in *Igf-1* and *Mstn* mRNA levels. IGF-1 and myostatin positively and negatively regulate myotube differentiation [30,39,40,41,42,43]. In Drosophila, the delivery of small interfering RNA (siRNA) against Mio/dChREBP into muscle resulted in flight defects due to altered myofibril shape and size [30]. Moreover, decreasing activity through the IGF-1 signaling pathway leads to muscle wasting and affects the genes encoding metabolic enzymes as well as macromolecule stores. Myostatin is the most well-known member of this superfamily in the field of muscle research because of the profound hypermuscularity of myostatin-KO mice [44]. The relationship between ChREBP and *Igf-1* or *Mstn* mRNA in muscle remain unclear, and further investigations are needed.

Our study revealed that muscle limb grip strength tended to decrease in WT mice. Protein restriction (a low-protein diet) is known to lead to muscle weight loss [5,6,7]. Lifelong protein restriction was shown to accelerate the loss of type-IIa muscle fibers and reduce muscle fiber size by impairing mitochondrial homeostasis and proteostasis at 18 months of age [45]. In humans, the prevalence of low muscle mass in Korean adults significantly increased with lower protein intake [46]. These findings suggest that a restricted protein intake may also promote muscle atrophy.

Moreover, both *Chrebp* deletion and low-protein diet feeding led to reduced muscle strength. Consistent with this finding, plasma gluconeogenic amino acid (glutamine and alanine) levels derived from muscle were highest in the low-protein diet-fed KO mice. This finding reflects the protein-sparing effect of carbohydrates [17,47,48,49]. The concept of “protein sparing” has been known since the 1940s [47,48,49]. In this original work, it was demonstrated that, with total fasting, a 70 kg man loses an amount of nitrogen equivalent to 80 g of protein per day [47,48]. Moreover, approximately half of these losses can be prevented by the administration of carbohydrates to fasted individuals. The maximal effect of glucose in preventing or sparing N losses is approximately 50% [47,48]. Moreover, some researchers have reported that starch intake is negatively associated with changes in urinary nitrogen excretion [48]. These findings suggest that carbohydrate intake may prevent muscle breakdown to maintain plasma glucose levels. Thus, *Chrebp* deletion and a low-protein diet resulted in an additive decrease in muscle mass and strength. Therefore, the simultaneous restriction of carbohydrate and protein intake may lead to a loss of muscle mass.

*Chrebp* deletion and a low-protein diet additively reduced muscle density, but would the effect on bone density be the same? Interestingly, *Chrebp* gene deletion led to an increase in bone mineral density. Consistent with these findings, the osteoblast surface area was significantly increased. Furthermore, consistent with the increase in this bone formation parameter, the level of *Osx* mRNA also increased, and the level of *Sost*, a Wnt signal inhibitor, decreased. With respect to bone absorption, the mRNA level of *Opg*, a modulator of RANKL, was increased. An epidemiological study revealed that a high glycemic index increases the risk of osteopenia and osteoporosis [50]. These findings suggest that *Chrebp* deletion may increase bone mineral density through bone formation rather than through bone absorption. Therefore, a higher intake of food with a low glycemic index (fruits and vegetables) may be a useful way to maintain bone mineral density.

In this study, a low-protein dietary intake decreased the bone mineral density despite increased bone formation and resorption in WT mice. Mild or short-term protein restriction reportedly may suppress bone resorption while transiently promoting bone formation due to secondary hyperparathyroidism [14]. When protein is restricted, bone resorption may predominate due to a lack of raw materials for collagen synthesis, which is necessary for bone formation [14]. In addition, protein deficiency may inhibit calcium absorption from the intestine, which mobilizes calcium from the bones to maintain blood calcium levels, and bone resorption may increase [14]. Therefore, mild protein restriction may lead to a decrease in bone mineral density by disturbing the balance between bone formation and resorption.

One limitation of the current study is that the interaction between bone and muscle was not considered. Mechanical and cytokine-mediated interactions between bone and muscle have been reported [14]. For example, exercise-induced IL6 acts on osteoblasts (and most likely osteocytes), leading to an increase in the production of RANKL, which in turn activates osteoclasts to acidify and remodel the bone matrix [51,52]. In our study, the bone *Rankl* mRNA levels were similar among the groups. After the sites of *Chrebp* mRNA expression in bone are identified, muscle-specific and osteocyte-specific knockout mice can be generated to elucidate the interactions between bone and muscle. Moreover, we could not detect the localization of the ChREBP protein in bone tissues, and whether *Chrebp* mRNA is expressed in osteoclasts or osteoblasts remains unclear. We detected *Chrebp* expression in bone tissues via immunohistochemistry, but we failed to detect *Chrebp* expression. To detect *Chrebp* mRNA in bone tissues, the isolation of osteoclasts and osteoblasts may be beneficial. Further investigations are needed to clarify the role of *Chrebp* in osteoclasts or osteoblasts via the use of tissue-specific *Chrebp* KO mice. Finally, the effect of Chrebp deletion on BMD may be indirect. In fact, body weight loss and decreased limb grip strength can decrease bone mineral density [32]. Moreover, visceral adipose tissue is negatively associated with bone mineral density [53,54]. Adiponectin, insulin/amylin/preptin, leptin, and adipocytic estrogens may be involved in the crosstalk between adipose tissue and bone tissue [55]. Recently, the concept of osteosarcopenia, the presence of osteopenia/osteoporosis and sarcopenia, has also been proposed [56]. These findings suggested that developing other mouse models with lower body weights, such as insulin deficiency and the administration of sodium glucose transport 2 inhibitors, may be beneficial.

## 5. Conclusions

In conclusion, to maintain muscle and bone function, adequate glucose and protein intake is important. Low-carbohydrate diets are often used for weight loss in individuals with obesity and other conditions. However, when protein restriction is added to the diet, it can cause not only weight loss but also the loss of muscle strength and bone density, as observed in this study with a low-protein diet and *Chrebp* deletion. To maintain bone and muscle mass, attention should be given to the protein–fat–carbohydrate ratio. This study highlights the need to be aware of both carbohydrate intake and protein intake. In addition, the detailed mechanism by which bone mineral density was reduced in the low-protein diet-loaded Chrebp knockout mice in the present study remains unclear. To elucidate the mechanism underlying osteosarcopenia caused by the crosstalk among fat, muscle, and bone tissues, an analysis of tissue-specific *Chrebp* knockout mice is needed.

## Figures and Tables

**Table 1:** List of primers used for semiquantitative real-time PCR.

Gene Name		Sequence
IGF1	F	CTGGACCAGAGACCCTTTGC
	R	GGACGGGGACTTCTGAGTCTT
Myostatin	F	CTGTAACCTTCCCAGGACCA
	R	TCTTTTGGGTGCGATAATCC
Glucokinase	F	CCGTGATCCGGGAAGAGAA
	R	GGGAAACCTGACAGGGATGAG
G6pc	F	TGGGCAAAATGGCAAGGA
	R	TCTGCCCCAGGAATCAAAAAT
Albumin	F	TGCTTTTTCCAGGGGTGTGTT
	R	TTACTTCCTGCACTAATTTGGCA
Prealbumin	F	TTGCCTCGCTGGACTGGTA
	R	TTACAGCCACGTCTACAGCAG
Col1a	F	CTTGCCAGCTTCCCCATCATCT
	R	CATGGGTCCTTCTGGTCCTCGT
BMP2	F	GTCGAAGCTCTCCCACTGAC
	R	CAGGAAGCTTTGGGAAACAG
Osx	F	AACTTCTTCTCCCGGGTGTG
	R	TGAGGAAGAAGCCCATTCAC
Sost	F	GTGTGATGTTGGGCTACGTG
	R	CCACCACAATCTCTCCCCTA
Rankl	F	AGGCTGGGCCAAGATCTCTA
	R	GTCTGTAGGTACGCTTCCCG
Opg	F	AGCAGGAGTGCAACCGCACC
	R	TTCCAGCTTGCACCACGCCG

## Data Availability

Some or all datasets generated during and/or analyzed during the current study are not publicly available but are available from the corresponding author upon reasonable request.

## Abbreviations

The following abbreviations are used in this manuscript:

MDPI	Multidisciplinary Digital Publishing Institute
DOAJ	Directory of open access journals
TLA	Three letter acronym
LD	Linear dichroism
